# Biomedical findings from NASA’s Project Mercury: a case series

**DOI:** 10.1038/s41526-018-0040-5

**Published:** 2018-03-13

**Authors:** William R. Carpentier, John B. Charles, Mark Shelhamer, Amanda S. Hackler, Tracy L. Johnson, Catherine M. M. Domingo, Jeffrey P. Sutton, Graham B. I. Scott, Virginia E. Wotring

**Affiliations:** 1 NASA Johnson Space Center, Houston, TX USA; 2 NASA Human Research Program, Houston, TX USA; 3 The Johns Hopkins University School of Medicine, Baltimore, MD USA; 4 Universities Space Research Association, Houston, TX USA; 5 National Space Biomedical Research Institute, Houston, TX USA; 6 Center for Space Medicine, Baylor College of Medicine, Houston, TX USA; 7 Department of Molecular and Cellular Biology, Baylor College of Medicine, Houston, TX USA; 8 Department of Pharmacology and Chemical Biology, Baylor College of Medicine, Houston, TX USA

## Abstract

The United States first sent humans into space during six flights of Project Mercury from May 1961 to May 1963. These flights were brief, with durations ranging from about 15 min to just over 34 h. A primary purpose of the project was to determine if humans could perform meaningful tasks while in space. This was supported by a series of biomedical measurements on each astronaut before, during (when feasible), and after flight to document the effects of exposure to the spaceflight environment. While almost all of the data presented here have been published in technical reports, this is the first integrated summary of the main results. One unexpected finding emerges: the major physiological changes associated with these short-term spaceflights are correlated more strongly with time spent by the astronaut in a spacesuit than with time spent in space per se. Thus, exposure to the direct stressors of short-duration (up to 34 h) spaceflight was not the dominant factor influencing human health and performance. This is relevant to current spaceflight programs and especially to upcoming commercial flights in which time spent in space (as on a suborbital flight) will be minor compared to the time spent in associated preparation, ascent, and return.

## Introduction

The launch of Sputnik I by the Soviet Union in October 1957 spurred the creation of Project Mercury in 1958. The project involved a series of one-man space missions of American astronauts into suborbital space and ultimately into low Earth orbit. The National Aeronautics and Space Administration (NASA) was charged with conducting these missions, which were among the first opportunities to observe the physiological impact on the human body to prolonged exposure to spaceflight environment factors.^1–3^

NASA selected seven active-duty military test pilots for temporary duty as astronauts.^4^ This ensured that individuals had the requisite technical skills and security clearances, excellent physical fitness, psychological capabilities for effective performance under extreme duress, and the ability to work productively both independently and within a team.^5^ At the time of flight, the Mercury astronauts were a homogeneous group: they were between 35 and 40 years of age, and due to the size constraints of the space capsule, stood no taller than 180 cm (5 feet, 11 inches).^1,3,5^

**Fig. 1:**
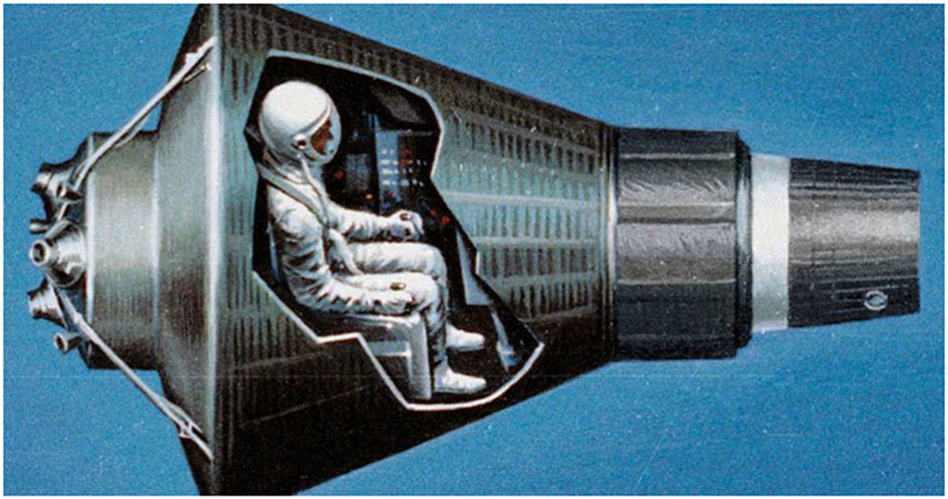
Mercury capsule diagram. This cutaway diagram shows that the capsule provided close quarters for its single occupant. Image modified from NASA original

Each Mercury astronaut was fitted with a specialized spacesuit intended as a back-up to the spacecraft’s life-support system. The suit had a dual-layer pressure bladder covered with nylon and weighed approximately 9.1 kg (20 pounds). Joint reinforcements and an aluminum-coated outer layer were added to improve mobility and thermal control, respectively. A hose connected to a fitting at the waist allowed oxygen to flow through the suit for cooling and then into the helmet for respiration. The astronauts fit snuggly into the conical Mercury vehicle that measured 2 m in height by 1.9 m in diameter at the base.^6^ Constrained in a small cabin (measuring approximately 1.73 m^3^), astronauts were restrained by a harness in a semi-supine posture (hips and knees flexed 90°) for the duration of their flights^3,6,7^ (see Fig. 1).

One of the greatest challenges in Project Mercury was characterizing both the physiological and psychological reactions that could occur in space. Space medicine was considered an extension of aviation medicine whose experts were consulted on the health risks of the space environment.^5^ Scientists knew little about human tolerance to a sustained weightless environment and some believed that weightlessness could lead to circulatory failure, disorientation, gastrointestinal and urinary disturbances, and muscular incoordination.^2,5^ Exposure to radiation was also a concern.

## Results

Most physiological measurements made during Project Mercury were easily implemented, common clinical measures for the purposes of medical monitoring during the missions. As a result of operational considerations, most data that were used for this analysis were collected inconsistently across flights. However, except for blood pressure, the in-flight vital sign measurements were reasonably consistent. The post-flight evaluations were not consistent in timing or in body posture. Specific in-flight tests varied between the flights.

Nevertheless, several findings were common to all missions. The key biomedical conclusion of Project Mercury was that human beings could function in the space environment for incrementally increasing flight durations of more than a day. Other major findings include the following:

### In-flight

- Heart rates of the astronauts were generally increased compared to those measured during centrifuge runs. Changes in heart rates from weightlessness to reentry and during planned exercise tests (a series of pulls on an elastic bungee cord) were within the same range as observed during simulations.
- Respiration rates were higher at lift-off for most crewmembers but during flight and reentry were similar to simulations.
- In-flight blood pressure was similar to that seen during pre-flight simulations.
- All astronauts reported that sensory functions seemed normal during their missions. They reported no changes in vision, hearing, vestibular function, taste, or smell.
- All astronauts reported that chewing and swallowing of liquids and solid foods was normal.
- Urination was also normal.

### Post-flight

- Body temperature was slightly increased above the pre-flight measurement in all flights (Table 1).
- Increased heart rate was noted following all Mercury flights (Table 1).
- Weight loss was noted following all Mercury flights (Table 2).
- Systolic blood pressure was lower in five of the six astronauts when measured in the same body position. .
- Estimated fluid intake from the time of the pre-flight to the post-flight physical examination varied widely from 240 mL to 2303 mL, at an average rate of 27.4 to 151.5 mL/h.
- Urine output varied widely from 295 to 2360 mL and was strongly associated with fluid intake, and flow rate varied from an average of 47.6 to 153.3 mL/h.

**Table 1:** Pre/post body temperature and heart rates

Flight	Temperature (°F)			Heart rate (bpm)		
	Pre	Post	Change	Pre	Post	Change
MR-3	99.0	100.2	1.2	68	76	8
MR-4	97.8	100.4	2.6	68	90	22
MA-6	98.2	99.2	1.0	68	76	8
MA-7	97.2	97.6	0.4	60	78	18
MA-8	97.6	99.4	1.8	72	92	20
MA-9	97.4	99.4	2.0	76	86	10

**Table 2:** Pre/post weight loss, actual and percentage

Flight	Weight (kg)			% Weight loss
	Pre	Post	Change	
MR-3	76.79	75.70	−1.09	1.42
MR-4	68.27	66.80	−1.47	2.15
MA-6	77.79	75.30	−2.49	3.20
MA-7	69.85	67.10	−2.75	3.94
MA-8	80.19	78.20	−1.99	2.48
MA-9	66.68	63.20	−3.48	5.22

### Loss of body mass

**Fig. 2:**
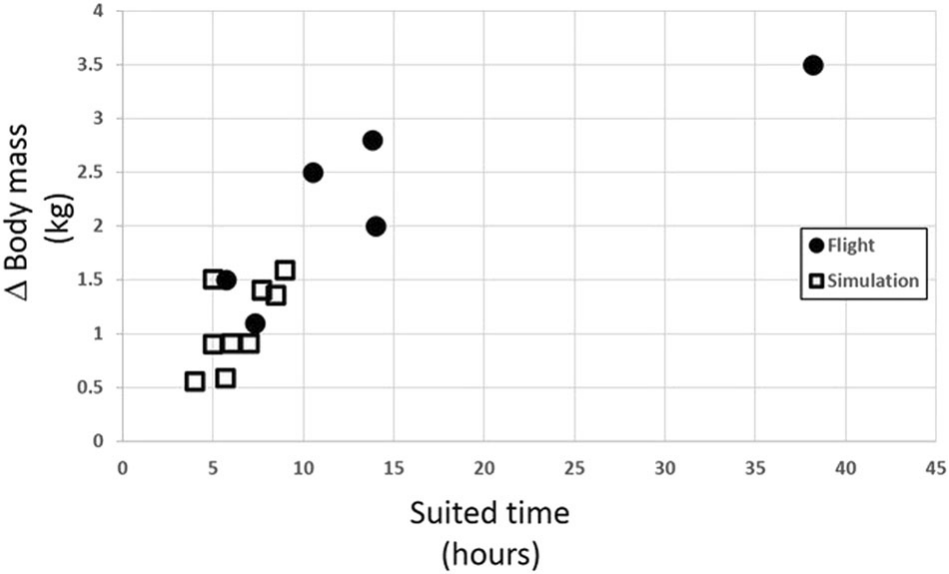
Changes in body mass correlate with time in the spacesuit. Body mass data from all Project Mercury flights and simulations show a correlation with time spent in the suit. Since the weight losses observed in simulations (open squares) show the same trend as those observed during actual spaceflights (filled circles), weight loss was likely associated with time spent in the suit, that was common to both scenarios

All Mercury astronauts had a recorded weight loss ranging from 1.1 to 3.5 kg, or 1.4 to 5.2% of body weight. The linear relationships of weight loss to time spent in weightlessness (*r* = 0.792; *p* = 0.60) and to total flight time (*r* = 0.794; *p* = 0.059) are nearly identical. The two suborbital missions had flight durations of only 15 min with a 5-min period of weightlessness, but their pilots lost 1.1 to 1.5 kg (1.4–2.2% of body weight). There is a strong linear correlation between the weight loss time spent in the Mercury full pressure suit (Fig. 2, filled circles, *r* = 0.84; *p* = 0.036). Time in the full pressure suit is defined as starting with the donning of the spacesuit on launch morning and extending to the time of the first medical exam after the suit was removed. There is an even higher correlation when time in the spacesuit is compared to percent of total body weight loss (*r* = 0.87; *p* = 0.025). Crewmembers experienced similar weight losses following centrifuge runs, and following simulations in the spacecraft and in the procedures trainer while wearing Mercury full pressure suits (Fig. 2, open squares), supporting the notion that weight losses were related to suited time, rather than weightless time.

In simulations, the average rate of weight loss in a full pressure suit was 180 ± 55 g/h. The average rate of weight loss for flights with similar time in the pressure suit (MR-3 to MA-6) was 212 ± 55 g/h and for all flights (MR-3 to MA-9) was 179 ± 69 g/h.

### Fluid balance

Urine volume was measured for four flights: MR-4, MA-6, MA-7, and MA-9. The rate of excretion during the collection period varied widely from an average of approximately 30 mL/h for the MA-9 pilot to an average of 155 mL/h for the MA-7 pilot. Fluid intake during these flights varied from an average of 27.4 mL/h for the MA-6 pilot to 151.5 mL/h for the MA-7 pilot. Urine output during the collection period appeared to be directly associated with fluid intake.

The MA-7 pilot had a post-flight follow-up examination at approximately 11 h after landing during which a weight gain of 1.7 kg or approximately 155 g/h was observed, which is likely due primarily to fluid replacement. Fluid losses can have measurable effects on the cardiovascular system, as discussed below.

### Heart rate

All post-flight heart rates were increased compared to pre-flight measurements made in the same posture. Only sitting heart rates were available for all six Mercury astronauts; supine and standing rates were infrequent. Cardiovascular changes, including increased heart rate with change in position and the associated decreases in blood pressure, appear to be related to the weight loss. The rate of change in sitting heart rate (% increase in beats/h) was significantly correlated with the rate of change in weight (% decrease in kg/h) for time spent in the spacesuit (*r* = 0.85; *p* = 0.03).

**Fig. 3:**
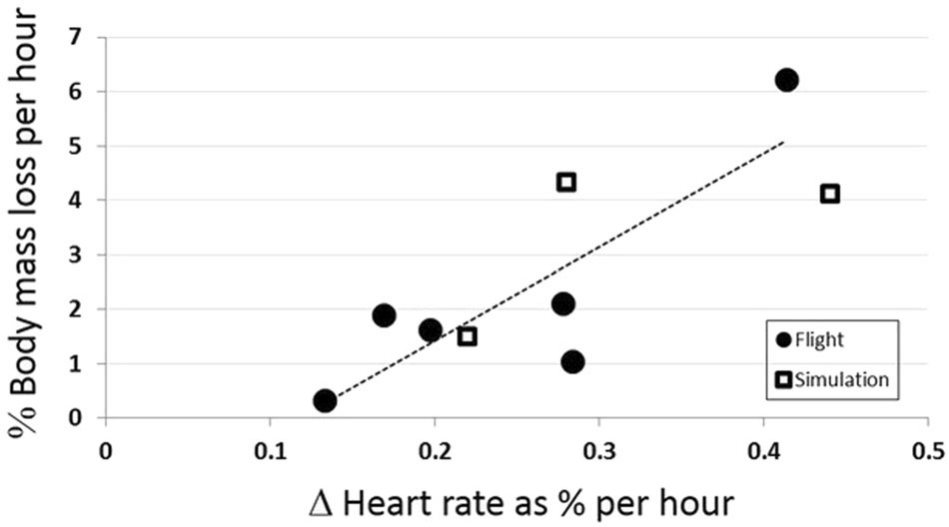
Heart rate elevations during missions correlated with the rate of body mass loss. Both variables are shown as a percentage: (pre-flight–post-flight) divided by pre-flight. Linear regression of flight data (dashed line) had a correlation coefficient of 0.72; note that simulation data, although few in number, also appear to fit this correlation

Heart rate increases were also recorded following one MA-8 and two MR-4 simulations. These heart rate increases were also associated with weight losses and were in a similar range as post-flight (Fig. 3).

### Blood pressure

Post-flight blood pressure measurements were less standardized than heart rate measurements in terms of timing and body position. Of the five astronauts who had pre-flight sitting blood pressures recorded, only three had post-flight sitting measurements. One astronaut had pre-flight and post-flight supine measurements and one had standing measurements. In all but one case, post-flight systolic pressure measurements in similar body positions were lower than pre-flight values by 3–18 mmHg. Systolic pressure was also lower following four out of five simulations. In general, a greater decrease in systolic blood pressure was associated with a higher increase in heart rate.

Compared to the changes in systolic pressure, the post-flight diastolic pressures were much more variable. However, calculated pulse pressure was lower in four of the five astronaut measurements, but body position during measurements was not consistent. Mean arterial pressure was lower in all cases, due mainly to the decrease in systolic pressure. The MA-8 pilot had a drop in standing systolic pressure compared to his supine systolic pressure. This was associated with a significant increase in standing heart rate. The MA-9 pilot had a low systolic pressure while supine in the spacecraft and he became presyncopal while standing after egressing. His supine rest and tilt systolic and diastolic pressures were significantly decreased post-flight. Pulse pressure was narrowed and mean arterial pressure was decreased. These findings are consistent with orthostatic hypotension. His weight gain that may be explained by fluid replacement was also associated with a return to normal pre-flight blood pressure.

## Discussion

The six Mercury astronauts were placed into the essentially unknown and potentially dangerous environment of space. No established normal physiological values for spaceflight stress parameters existed at the time, nor were there proven methods for determining whether an astronaut was approaching a threshold of tolerance. Personalized physiological norms needed to be empirically derived for each astronaut and used to evaluate the in-flight status of each individual. These norms, which included both physiological factors and clinical assessments, would be based on measurements made before, during, and following training trials on centrifuges and flight simulators. Project Mercury demonstrated that humans could function during spaceflight of up to 34 h without notable deterioration of normal body functions or significant degradation of pilot function.

Decreased appetite was reported for all four orbital flights and water intake was also reduced during three of the four flights. Weight loss of the magnitude occurring over the short duration of these spaceflights, especially the suborbital flights, is likely due to a combination of limited intake, loss from the skin and respiratory tract, and increased sweating while wearing the Mercury full pressure suit. Individual data suggest that the observed weight loss may have been be reduced or prevented by a replacement intake that includes fluid, electrolytes, and nutrients. The loss of water through the skin is obligatory in most environments and the need to dissipate body heat through sweating takes precedence over conserving water, which can result in dehydration. The evaporation of moisture from the skin serves as a primary method of cooling in a ventilated pressure suit. The estimate for average insensible and sweat loss for these four flights was 150 mL/h (range 96–194). When test subjects were evaluated seated and mildly active in full pressure suits during simulated flight conditions, sweat production of 300 to 600 mL/h was reported.

The Mercury spaceflights were brief relative to modern missions, but their durations are comparable to expected commercial tourist spaceflights. This new analysis spanning all the manned Mercury missions shows that certain physiological responses associated with spaceflight correlate better with suited time than with time in weightlessness or time in flight. Since operational aspects like spacesuits can be modified to reduce their physiological impact, this finding can be used to improve crew health, comfort, and performance on future spaceflights.

## Methods

**Fig. 4:**
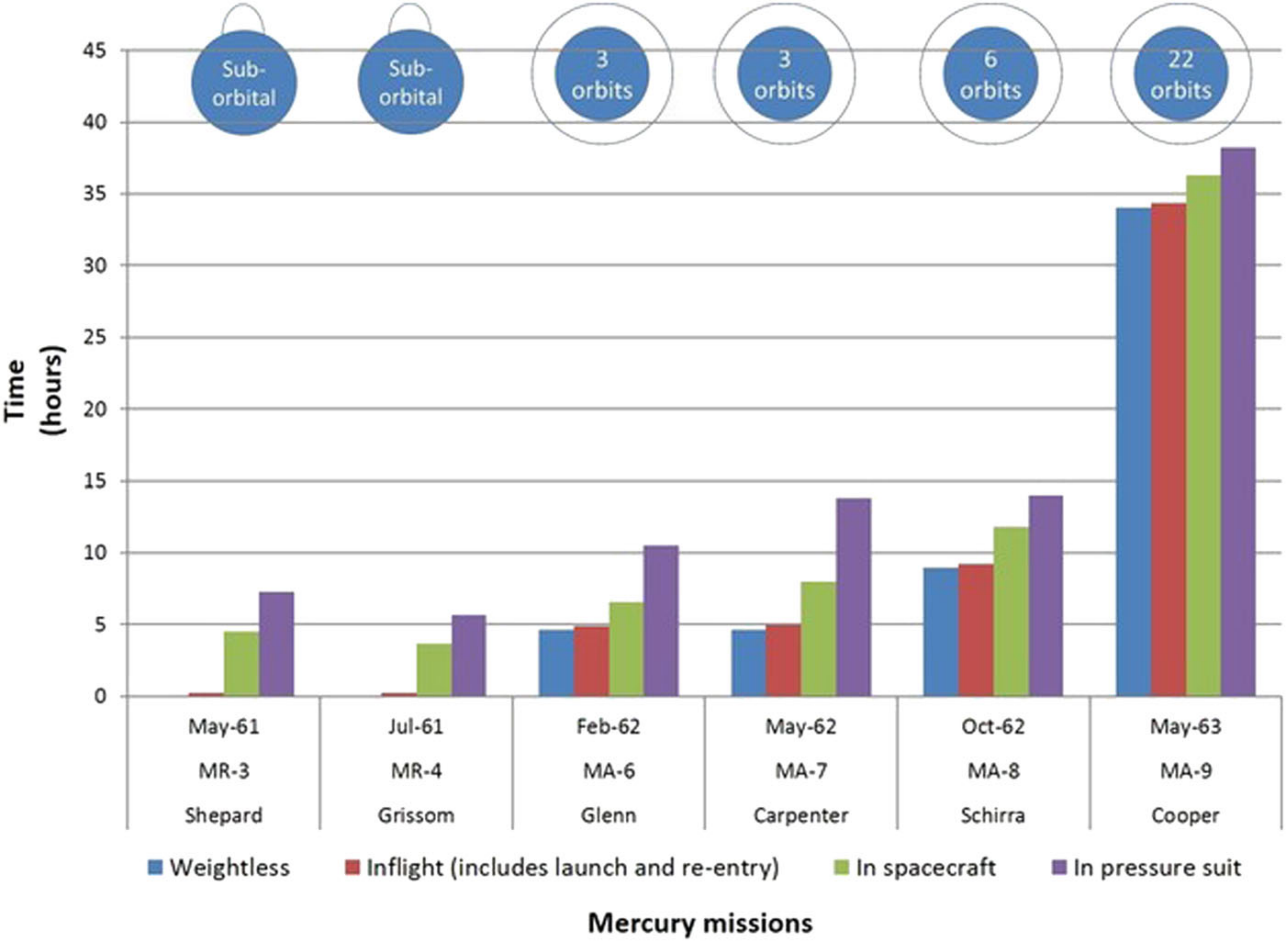
Mission architecture for Project Mercury. Histogram shows time in hours spent in weightlessness (blue), in-flight (red), in the spacecraft (green), and in the pressure suit (purple). Note that suit time did not differ greatly for the first 5 missions, although weightless time did. Icons (top) indicate suborbital flights and number of orbits for each orbital flight

Flights were designated and numbered sequentially according to the launch vehicle: the suborbital missions used the Redstone rocket and were designated “MR” for “Mercury-Redstone”; the orbital missions employed the Atlas rocket and were designated “MA” for “Mercury-Atlas.” Technical characteristics of the six crewed Mercury flights (MR-3, MR-4, MA-6, MA-7, MA-8, and MA-9) are shown in Table 3. Mission designs and time spent in the pressure suit, in the spacecraft, in-flight, and in weightlessness are shown in Fig. 4. Other flights in Project Mercury are not discussed further in this paper, including seven unmanned test flights and two chimpanzee flights to demonstrate the compatibility of complex life with the spacecraft and flight environment. These subjects were the first Americans to experience weightlessness; their data were collected for medical monitoring purposes. The analysis presented here is new, but the data have been published previously (Table 4).

**Table 3:** Project Mercury missions

Mission	Date	Flight duration (h:min:s)	Weightless time (h:min:s)	# Earth orbits	Pilot
MR-3	5/5/1961	15:28	5:04	0	A.B. Shepard
MR-4	7/21/1961	15:37	5:00	0	V.I. Grissom
MA-6	2/20/1962	4:55:23	4:38:00	3	J.H. Glenn
MA-7	5/24/1962	4:56:05	4:39:00	3	M.S. Carpenter
MA-8	10/3/1962	9:13:11	8:56:22	6	W.M. Schirra
MA-9	5/15/1963	34:19:49	34:03:30	22	L.G. Cooper

**Table 4:** Pre/post Hct and Hb

Flight	Hct (%)			Hb (g/100 ml)		
	Pre (mean)	Post	Change	Pre (mean)	Post	Change
MR-3	45.0	40.0	−5.0	13.0	13.5	0.5
MR-4	42.5	42.2	−0.3	14.1	14.4	0.3
MA-6	42.3	46.0	3.7	14.3	16.1	1.8
MA-7	44.5	50.0	5.5	14.4	16.0	1.6
MA-8	44.0	47.0	3.0	15.0	14.5	−0.5
MA-9	44.3	49.0	4.7	14.8	16.5	1.7
Mean	43.77	45.70	1.93	14.27	15.17	0.90
SD	1.11	3.90	3.94	0.70	1.20	0.94

*Hct* hematocrit, * Hb* hemoglobin

These missions predate the Privacy Act of 1974 and Institutional Review Board (IRB), so data collection and analysis protocols were not approved by any such boards; however, the authors received a determination from the NASA IRB Chairman that this current analysis did not require IRB review.

## Measurements

Biomedical measurements were initially limited to the most basic vital signs, but additional measures were added as missions grew longer. Flight surgeons relied on sensors that measured body core temperature, respiration rate, and electrical activity in the heart,^8–10^ to aid in the real-time assessment of astronaut status. Measuring physiological responses in pilots was not new, but such prolonged monitoring had not been previously attempted. Along with remote monitoring, verbal feedback from the astronauts was vital to determining their well-being, but, like the monitoring, could only be employed when in range of one of the limited number of ground stations.^5^

### Body temperature

Body temperature was measured rectally during all but the final Mercury mission, to give the most effective temperature indication with the simplest device.^11^ On the last flight, MA-9, which was much longer than previous missions, the thermistor was modified for oral use for reasons of comfort.^2^

### Blood pressure

A blood pressure system was not technically feasible at the beginning of Project Mercury but was developed in time for use on the four orbital missions.^12^ With continued refinement, the last two missions, MA-8 and MA-9, returned excellent blood pressure data.^2,13^ Body postures were not consistent across measurements; for approximately half the subjects, posture was the same at both pre-flight and post-flight measurements.

### Respiration

A thermistor sensing system which measured air movement directly and eliminated the need for chest straps was used for the first four flights.^11^ Because the thermistor responded to ambient temperature, it was heated to a temperature above the surroundings so respiratory airflow could be measured by the cooling of the probe.^8^ An impedance pneumograph system was implemented for the final two flights which provided accurate respiration information during most of the flight.^5^

### Cardiac electrical activity

An electrocardiogram electrode was developed for Project Mercury that provided good electrical contact with the subject’s skin, easy application, no physical interference with the subject, resistance compatible with the amplifier system, and the capacity to function for 30+ h of flight.^5,8^ This solution gave distinguishable QRS complexes and T-waves and was used throughout the project.^8^

### Body weight

Weight was measured before and after flight.^11^ All weight measurements were made nude with an empty bladder on a beam-balance scale. Pre-flight measurements were made on the same scale by the same operators, while post-flight measurements were made on different scales by different operators on different recovery ships. These scales measured in pounds interpolated to the nearest quarter pound by the operator; values given in this report have been converted to metric units. While the elapsed time between the pre-flight physical examination and launch was relatively consistent at 2.5 to 4.2 h, the elapsed time between landing and the first post-flight medical examination varied from 30 min to 4.6 h.^2,11–15^

### Urine collection

Urine was collected using an in-suit device for all flights but the first one. The start time for the collection could not be documented but was estimated to be after the launch day physical examination and prior to donning the suit. The urine volume was measured following suit removal post-flight and included urinations on the launch pad, during flight, and post-flight.^2,12,14,15^ Thus, urine collection time was somewhat longer than suited time. For the longest flight, the collection bag could be emptied into storage bags aboard the spacecraft using a syringe-type pump. The urine collection device attachment failed during landing on the MA-8 flight and most of the collection was lost,^13^ but accurate measurements were made for the other four flights.

### Insensible Fluid Loss and Sweat Loss

Insensible fluid loss and sweat loss together were estimated as that portion of total weight loss that equals the pre-flight weight plus subsequent pre-flight and in-flight fluid intake minus urine volume.

### Data availability statement

The data analyzed are available online in the NASA History Office (https://history.nasa.gov/) or on the NASA Technical Reports Server (https://ntrs.nasa.gov/). Our collected data have been submitted for archiving in NASA Lifetime Surveillance of Astronaut Health (https://lsda.jsc.nasa.gov/lsah_home1.aspx).
